# The Role of Adenosine A1 and A2A Receptors in the Caffeine Effect on MDMA-Induced DA and 5-HT Release in the Mouse Striatum

**DOI:** 10.1007/s12640-014-9501-0

**Published:** 2014-11-13

**Authors:** A. M. Górska, K. Gołembiowska

**Affiliations:** Institute of Pharmacology, Polish Academy of Sciences, Smętna 12, 31-343 Kraków, Poland

**Keywords:** MDMA, Caffeine, DA, 5-HT, Microdialysis, Mouse

## Abstract

3,4-Methylenedioxymethamphetamine (MDMA, “ecstasy”) popular as a designer drug is often used with caffeine to gain a stronger stimulant effect. MDMA induces 5-HT and DA release by interaction with monoamine transporters. Co-administration of caffeine and MDMA may aggravate MDMA-induced toxic effects on DA and 5-HT terminals. In the present study, we determined whether caffeine influences DA and 5-HT release induced by MDMA. We also tried to find out if adenosine A1 and A2A receptors play a role in the effect of caffeine by investigating the effect of the selective adenosine A1 and A2A receptor antagonists, DPCPX and KW 6002 on DA and 5-HT release induced by MDMA. Mice were treated with caffeine (10 mg/kg) and MDMA (20 or 40 mg/kg) alone or in combination. DA and 5-HT release in the mouse striatum was measured using in vivo microdialysis. Caffeine exacerbated the effect of MDMA on DA and 5-HT release. DPCPX or KW 6002 co-administered with MDMA had similar influence as caffeine, but KW 6002 was more potent than caffeine or DPCPX. To exclude the contribution of MAO inhibition by caffeine in the caffeine effect on MDMA-induced increase in DA and 5-HT, we also tested the effect of the nonxanthine adenosine receptor antagonist CGS 15943A lacking properties of MAO activity modification. Our findings indicate that adenosine A1 and A2A receptor blockade may account for the caffeine-induced exacerbation of the MDMA effect on DA and 5-HT release and may aggravate MDMA toxicity.

## Introduction

3,4-Methylenedioxymethamphetamine (MDMA, “ecstasy”) is a designer drug structurally related to the hallucinogenic mescaline and amphetamine. Its illicit use by “rave” party participants is a serious social problem. In addition, it induces neurotoxicity observed in experimental models and in humans. The data obtained in laboratory animals in vivo have revealed that MDMA interacts with monoamine transporters to stimulate non-exocytotic release of serotonin (5-HT), dopamine (DA), and noradrenaline (NA) (Baumann et al. 2005; Gudelsky and Nash 1996; Sulzer et al. 2005; Yamamoto and Spanos 1988). MDMA has mood-enhancing properties and hallucinogenic effects in humans (Sulzer et al. 2005). Its acute peripheral symptoms include hyperthermia, increased blood pressure, tachycardia, acute renal and liver failure, convulsions, and cerebral hemorrhage resulting in death (Capela et al. 2009). A long-term MDMA intake causes neurotoxic effects to the serotonergic fibers in the forebrain leaving raphe cell bodies unaffected (Xie et al. 2006) as observed in rats and non-human primates (Capela et al. 2009). A wide variety of abused drugs are often found in ecstasy tablets to gain a stronger stimulant effect and such combinations of MDMA with other compounds may be extremely toxic leading to enhanced adverse effects. For instance, high amount of caffeine has been often detected in ecstasy tablets. Individuals exposed to excessive doses of caffeine presented anxiety, agitation, hallucinations, convulsions, and mimicking the effects of stimulant recreational drugs (Davies et al. 2012). The primary action of caffeine is to block adenosine A1 and A2A receptors which leads to secondary effects on many classes of neurotransmitters (Fredholm et al. 1999). Inhibitory adenosine A1 receptors are present in almost all brain areas and their stimulation can suppress neuronal excitability (Fredholm et al. 1994). Adenosine A2A receptors concentrated in the dopamine rich areas of the brain activate adenylyl cyclase and some types of voltage-sensitive Ca^2+^-channels (Fredholm et al. 1994). Thus, adenosine A1 and A2A receptors have opposing actions at cellular and neuronal levels. The central stimulatory effect of caffeine seems to be related with the blockade of adenosine A1 receptors causing increases of 5-HT, DA and NA turnover (Hadfield and Milio 1989), elevation of DA level in the striatum (Morgan and Vestal 1989). In addition, an A1 antagonist was shown to enhance locomotion in rodents (Popoli et al. 1996). A2A receptors are abundant in the striatum and nucleus accumbens where they are expressed on the GABAergic neurons or are present on glutamatergic neuronal terminals thus controlling the basal ganglia output and input neurons (Svenningsson et al. 1998). There is evidence that A2A receptors oppose the effects of dopamine D2 receptors (Ferré et al. 1997). Thus, an inhibition of A2A receptors by caffeine can increase rotation behavior induced by dopamine agonists (Fenu et al. 1997), while dopamine receptor antagonists can inhibit the stimulatory effects of caffeine on locomotion (Garret and Holtzman 1994). Caffeine co-administered with MDMA potentiated the MDMA effect on extracellular DA level in the striatum of anesthetized rats (Ikeda et al. 2011) and enhanced MDMA-induced DA release from the rat striatal slices and this effect was suggested to be mediated via adenosine A1 receptors (Vanattou-Saïfoudine et al. 2011). On the other hand, exacerbation of MDMA-induced hyperthermia by caffeine is proposed to result from the inhibition of adenosine A2A receptors (Vanattou-Saïfoudine et al. 2010). Hyperthermia and neuroinflammation after acute but not chronic administration of caffeine and MDMA have been reported to cause neurotoxic effects in rodents (Khairnar et al. 2010; McNamara et al. 2006; Ruiz-Medina et al. 2013; Vanattou-Saïfoudine et al. 2011). In contrast to rats and non-human primates, MDMA produces rather dopaminergic neurotoxicity in mice (Colado et al. 2001). However, depending on MDMA dosage and mouse strain, it can cause 5-HT neurotoxicity, as well (Fornai et al. 2004). According to the hypothesis of Sprague et al. (1998), acute doses of MDMA induce 5-HT and DA release. 5-HT released by MDMA decreases inhibitory GABAergic transmission via 5-HT2A/C receptors situated on GABA interneurons, this effect is followed by increased DA release. The excessive DA transported into serotonergic terminals may be metabolized through MAO-B into 3,4-dihydroxyphenylacetic acid (DOPAC) and hydrogen peroxide which would result in free radical generation. Thus, either DA or 5-HT may be a determinant of MDMA neurotoxicity. Co-administration of caffeine and MDMA may aggravate MDMA-induced toxic effects on DA and 5-HT terminals via the mechanism engaging adenosine A1/A2A receptors. To date, there are no data about in vivo effects of caffeine and MDMA co-administration in mice. Therefore, in our study, we were interested in investigating whether acute treatment with caffeine affects MDMA-induced release of DA and 5-HT in the mouse striatum using in vivo microdialysis. In addition, we attempted to explain the specific role of adenosine A1 and A2A receptors in the caffeine effect by investigating the influence of selective adenosine A1 and A2A receptor antagonists, 8-cyclopentyl-1,3-dipropylxanthine (DPCPX), and (E)-1,3-diethyl-8-(3,4-dimethoxystyryl)-7-methyl-3,7-dihydro-1H-purine-2,6-dione (KW 6002) on the MDMA-induced DA and 5-HT release. To exclude the contribution of MAO inhibition by caffeine in the caffeine effect on MDMA-induced increase in DA and 5-HT, we also tested the effect of the nonxanthine adenosine receptor antagonist CGS 15943A lacking properties of MAO activity modification.

## Materials and Methods

### Animals

Experiments were performed on adult male (8–10 weeks old) C57BL/6J inbred mice. The animals were housed 5–7 per cage, under a 12-h light/12-h dark cycle, with free access to standard food and tap water. The experiments were conducted in accordance with the European Union guidelines regarding the care and use of laboratory animals (Council Directive 86/609/EEC of November 24, 1986) and were approved by the II Local Bioethics Commission (Kraków, Poland).

### Drugs and Reagents

Caffeine, DPCPX (8-cyclopentyl-1,3-dipropylxanthine) and CGS 15943A (9-chloro-2-(2-furyl)[1,2,4]triazolo[1,5-c]quinazoline-5-amine) were obtained from Sigma-Aldrich (Poznań, Poland), while KW 6002 [(E)-1,3-diethyl-8-(3,4-dimethoxystyryl)-7-methyl-3,7-dihydro-1H-purine-2,6-dione] was purchased from Selleckchem (USA). Caffeine was dissolved in 0.9 % NaCl, while DPCPX and KW 6002 were dissolved initially in dimethyl sulfoxide (DMSO) and were then suspended in 0.3 % Tween 80. All injections were done with intraperitoneal route and control animals received respective vehicles. The chemicals used for HPLC were purchased from Merck (Warsaw, Poland).

### Brain Microdialysis

Animals were anesthetized with ketamine (7.5 mg/kg) and xylazine (1 mg/kg), and a vertical microdialysis probes were implanted into the striatum using the following coordinates: AP + 1.0, L + 1.8, V − 3.8 (Paxinos and Franklin 2001). On the next day, probe inlets were connected to a syringe pump (BAS, IN, USA) which delivered a CSF composed of [mM]: NaCl 147, KCl 2.7, MgCl_2_ 1.0, CaCl_2_ 1.2; pH 7.4 at a flow rate of 1.5 µl/min. After 1 h of the washout period, three basal dialysate samples were collected every 30 min; then animals were injected with appropriate drugs in doses indicated in figure captions and fraction collection continued for 270 min. At the end of the experiment, the mice were sacrificed and their brains were histologically examined to validate the probe placement.

### Analytical Procedure

DA, 5-HT, 3-MT, 3,4-dihydroxyphenylacetic acid (DOPAC), homovanillic acid (HVA), and 5-hydroxyindoleacetic acid (5-HIAA) were analyzed by high-performance liquid chromatography (HPLC) with coulochemical detection. Chromatography was performed using an Ultimate 3000 System (Dionex, USA), coulochemical detector Coulochem III (model 5300, ESA, USA) with 5020 guard cell, 5014B microdialysis cell and Hypersil Gold C18 analytical column (3 × 100 mm). The mobile phase was composed of 0.1 M potassium phosphate buffer adjusted to pH 3.6, 0.5 mM EDTA, 16 mg/L 1-octanesulfonic acid sodium salt, and 2 % methanol. The flow rate during analysis was set at 0.7 ml/min. The applied potential of a guard cell was +600 mV, while those of microdialysis cells were: *E*_1_ = −50 mV, *E*_2_ = +300 mV with a sensitivity set at 50 nA/V. The chromatographic data were processed by Chromeleon v. 6.80 (Dionex, USA) software run on a PC computer.

### Data Analysis

All obtained data were presented as a percent of the basal level assumed as 100 %. The statistical significance was calculated using a repeated measures ANOVA or where appropriate a one-way ANOVA, followed by Tukey’s post-hoc test. The results were considered statistically significant when *P* < 0.05.

## Results

### The Effect of Caffeine and Adenosine A1 and A2A Receptor Antagonists DPCPX and KW 6002 on DA Release Induced by MDMA in the Striatum of the Mouse

**Fig. 1:**
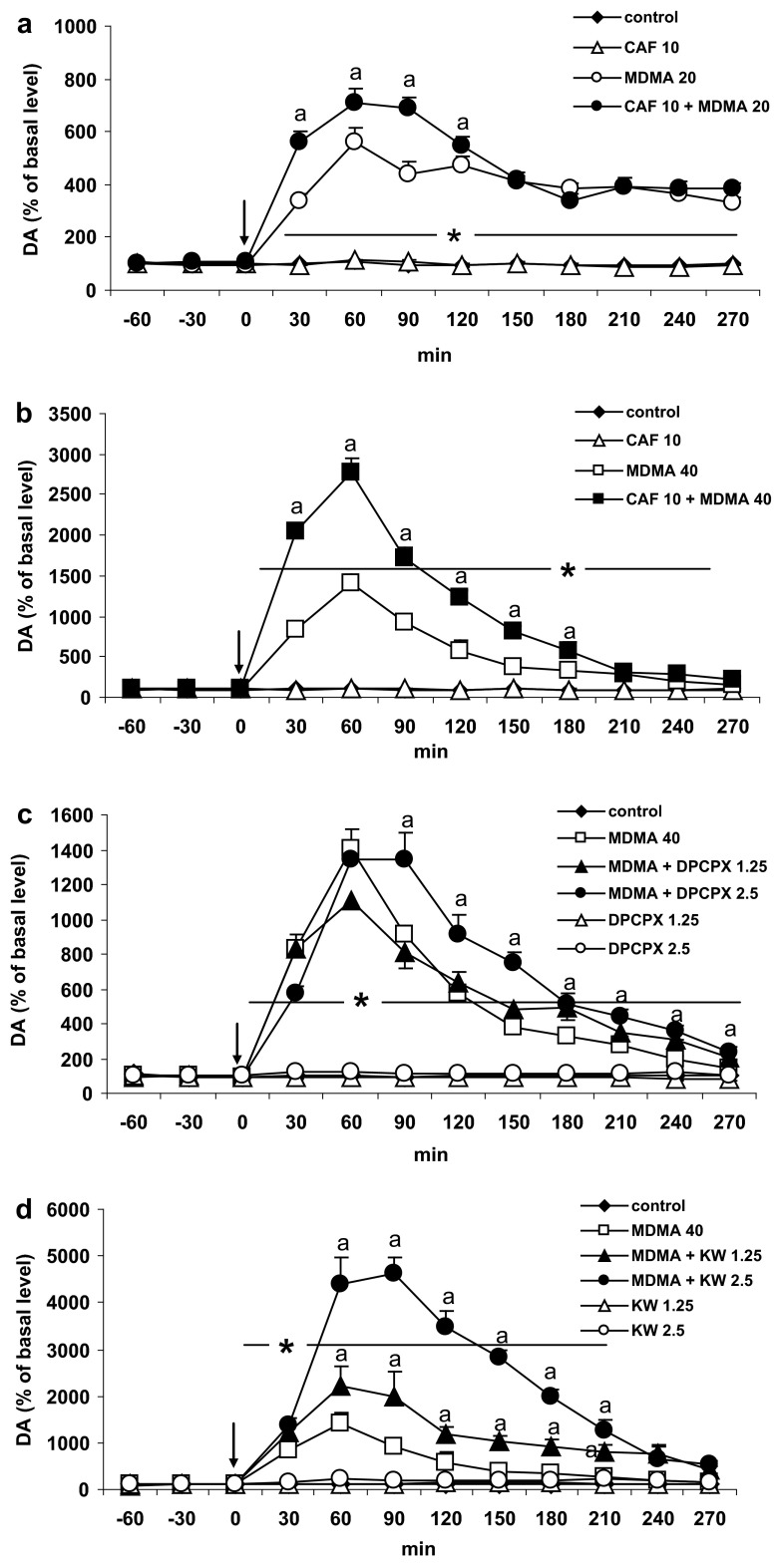
The effect of caffeine (CAF) and adenosine A1 and A2A receptor antagonists, DPCPX and KW 6002 (KW) on DA release induced by MDMA in the mouse striatum. CAF (10 mg/kg), DPCPX (1.25 and 2.5 mg/kg), and KW (1.25 and 2.5 mg/kg) were injected simultaneously with MDMA 20 or 40 mg/kg as indicated with an *arrow*. Values are the mean ± SEM (*n* = 6–8 animals). *P < 0.0001 represents a significant difference in comparison to control group; “*a*” *P* < 0.0002 represents a significant difference in comparison to MDMA group (repeated measures ANOVA and Tukey’s post-hoc test)

MDMA at doses of 20 and 40 mg/kg significantly (*P* = 0.0001) increased DA release by ca. 560 and 1,400 % of the basal level at 60 min after administration. Caffeine at a dose of 10 mg/kg, but not at a dose of 5 mg/kg (results not shown) markedly enhanced the effect of MDMA on DA release given at a dose of 40 mg/kg (*P* = 0.0002); however, it did not affect significantly the increase in DA release produced by MDMA in a lower dose of 20 mg/kg (Fig. 1a, b). Repeated measures ANOVA of these data showed a statistically significant effect of treatment groups [*F*_5,28_ = 262, *P* = 0], sampling period [*F*_8,224_ = 312, *P* = 0], and the interaction between treatment groups and sampling period [*F*_40,224_ = 122, *P* = 0].

The selective adenosine A1 receptor antagonist DPCPX at a dose of 2.5 mg/kg but not at a dose of 1.25 mg/kg significantly (*P* = 0.0001) enhanced the effect of MDMA (40 mg/kg) on DA release (Fig. 1c). Repeated measures ANOVA showed a significant effect of treatment groups [*F*_5,26_ = 445, *P* = 0], sampling period [*F*_8,208_ = 245, *P* = 0], and the interaction between treatment groups and sampling period [*F*_40,208_ = 62, *P* = 0].

The selective adenosine A2A receptor antagonist KW 6002 at both doses of 1.25 and 2.5 mg/kg significantly (*P* = 0.0001) increased the effect of MDMA (40 mg/kg) on DA release (Fig. 1d). Repeated measures ANOVA showed a significant effect of treatment groups [*F*_5,28_ = 627, *P* = 0], sampling period [*F*_8,224_ = 194, *P* = 0], and the interaction between treatment groups and sampling period [*F*_40,225_ = 78, *P* = 0].

The basal extracellular DA level in dialysate fractions from the mouse striatum was 7.74 ± 0.71 (pg/10 μl, *n* = 103) and no significant differences between experimental groups were observed.

### The Effect of Caffeine and Adenosine A1 and A2A Receptor Antagonists DPCPX and KW 6002 on 5-HT Release Induced by MDMA in the Striatum of the Mouse

**Fig. 2:**
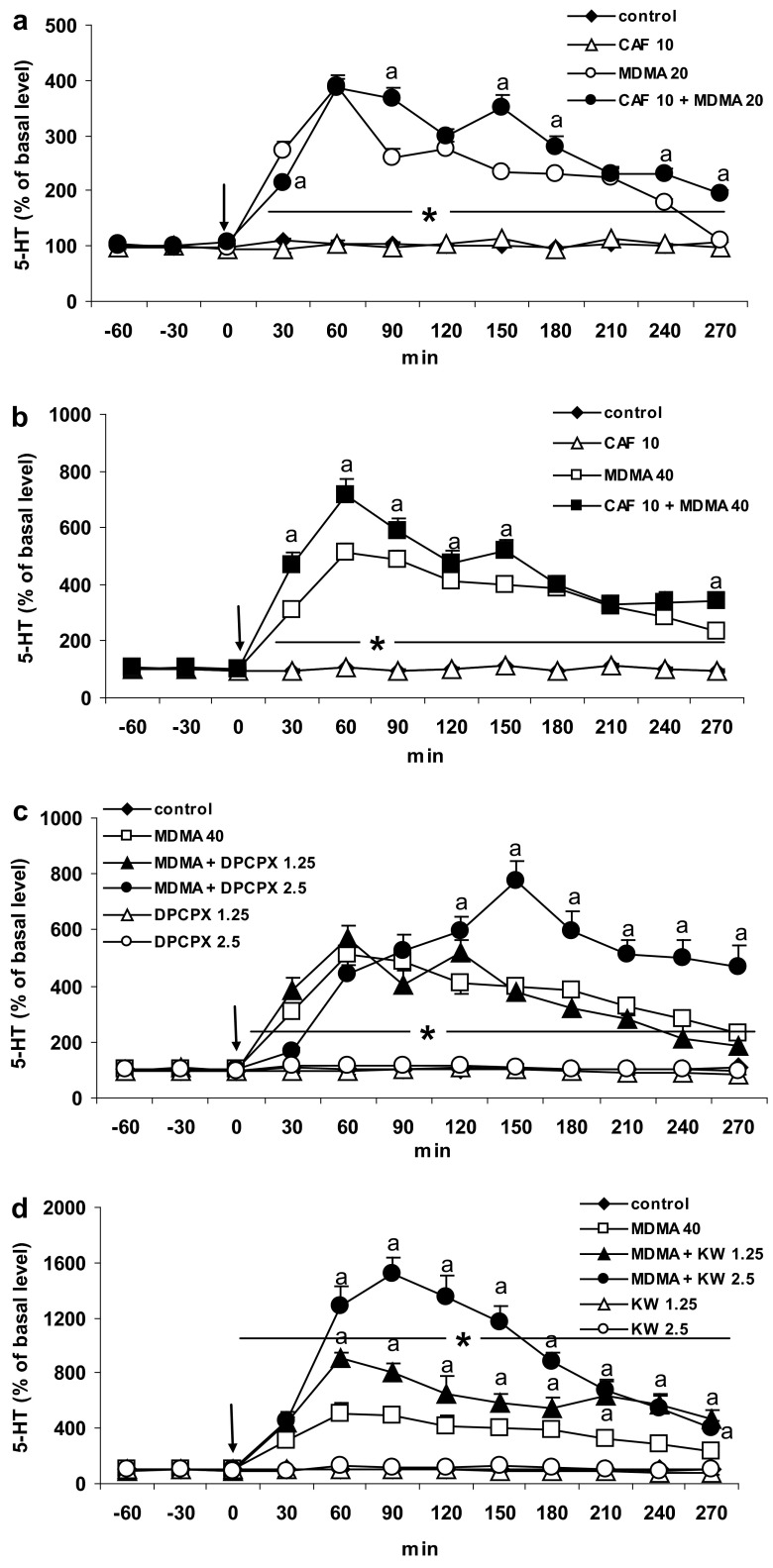
The effect of caffeine (CAF) and adenosine A1 and A2A receptor antagonists, DPCPX and KW 6002 (KW) on 5-HT release induced by MDMA in the mouse striatum. CAF (10 mg/kg), DPCPX (1.25 and 2.5 mg/kg), and KW (1.25 and 2.5 mg/kg) were injected simultaneously with MDMA 20 or 40 mg/kg as indicated with an *arrow*. Values are the mean ± SEM (*n* = 6–8 animals). *P < 0.0001 represents a significant difference in comparison to control group; “*a*” *P* < 0.0002–0.01 represents a significant difference in comparison to MDMA group (repeated measures ANOVA and Tukey’s post-hoc test)

MDMA at doses of 20 and 40 mg/kg significantly (*P* = 0.0001) increased 5-HT release in the mouse striatum by ca. 389 and 510 % of the basal level at 60 min after administration. Caffeine (10 mg/kg), but not at a dose of 5 mg/kg (results not shown) significantly increased the effect of both MDMA doses (20 and 40 mg/kg) on 5-HT release (*P* = 0.002 and *P* = 0.0001, respectively) (Fig. 2a, b). Repeated measures ANOVA showed a significant effect of treatment groups [*F*_5,26_ = 372, *P* = 0], sampling period [*F*_8,208_ = 101, *P* = 0], and the interaction between treatment groups and sampling period [*F*_40,208_ = 15, *P* = 0].

A selective adenosine A1 receptor antagonist DPCPX at a dose of 2.5 mg/kg but not 1.25 mg/kg significantly (*P* = 0.0001) enhanced the effect of MDMA (40 mg/kg) on 5-HT release (Fig. 2c). Repeated measures ANOVA showed a significant effect of treatment groups [*F*_5,23_ = 618, *P* = 0], sampling period [*F*_8,184_ = 80, *P* = 0], and the interaction between treatment groups and sampling period [*F*_40,184_ = 45, *P* = 0].

The selective adenosine A2A receptor antagonist KW 6002 at both doses of 1.25 and 2.5 mg/kg significantly (*P* = 0.0001) increased the effect of MDMA (40 mg/kg) on 5-HT release (Fig. 2d). Repeated measures ANOVA showed a significant effect of treatment groups [*F*_5,26_ = 236, *P* = 0], sampling period [*F*_8,208_ = 80, *P* = 0], and the interaction between treatment groups and sampling period [*F*_40,208_ = 34, *P* = 0]. The basal extracellular 5-HT level in dialysates from the mouse striatum was 2.30 ± 0.13 (pg/10 μl, *n* = 99) and no significant differences between experimental groups were observed.

### The Effect of Caffeine and Adenosine A1 and A2A Receptor Antagonists DPCPX and KW 6002 on MDMA-Induced Changes in Extracellular 3-MT Level in the Striatum of the Mouse

**Fig. 3:**
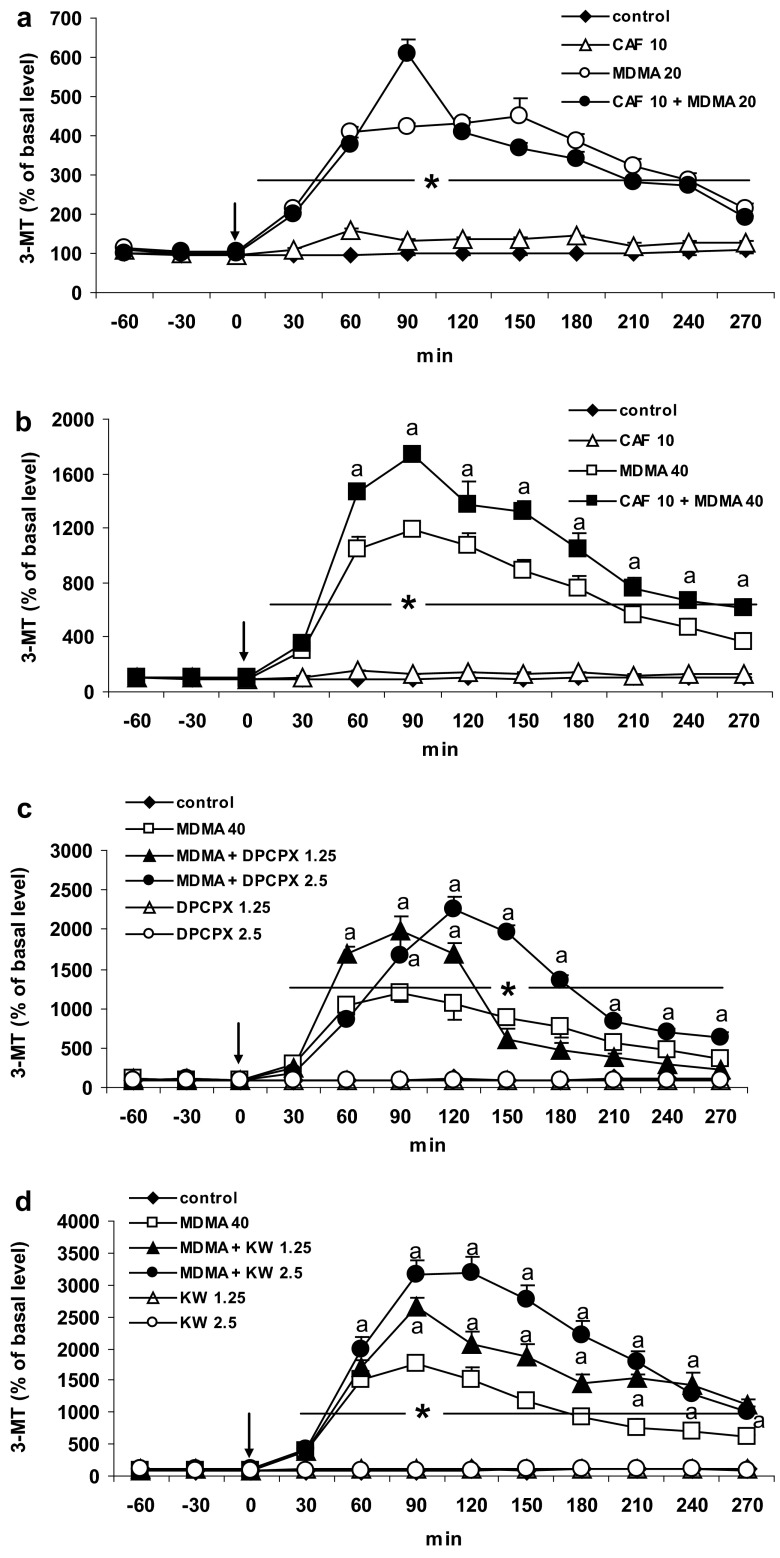
The effect of caffeine (CAF) and adenosine A1 and A2A receptor antagonists, DPCPX and KW 6002 (KW) on extracellular level of 3-MT increased by MDMA in the mouse striatum. CAF (10 mg/kg), DPCPX (1.25 and 2.5 mg/kg), and KW (1.25 and 2.5 mg/kg) were injected simultaneously with MDMA 20 or 40 mg/kg as indicated with an *arrow*. Values are the mean ± SEM (*n* = 6–8 animals). *P < 0.0001 represents a significant difference in comparison to control group; “*a*” *P* < 0.0002 represents a significant difference in comparison to MDMA group (repeated measures ANOVA and Tukey’s post-hoc test)

The level of the extraneuronal DA metabolite, 3-MT was significantly (*P* = 0.0001) increased by MDMA 20 and 40 mg/kg to ca. 711 and 1736 % of the basal values, respectively, between 60 and 120 min after administration. Caffeine (10 mg/kg) significantly (*P* = 0.0002) increased the effect of a higher dose (40 mg/kg) of MDMA on 3-MT extracellular level, but did not affect the increase in 3-MT induced by a lower dose of MDMA (Fig. 3a, b). Repeated measures ANOVA showed a significant effect of treatment groups [*F*_5,25_ = 274, *P* = 0], sampling period [*F*_8,200_ = 233, *P* = 0], and the interaction between treatment groups and sampling period [*F*_40,200_ = 57, *P* = 0].

The selective adenosine A1 receptor antagonist DPCPX at both doses, 1.25 and 2.5 mg/kg significantly (*P* = 0.0001 and *P* = 0.01, respectively) increased the effect of MDMA (40 mg/kg) on extracellular 3-MT level (Fig. 3c). Repeated measures ANOVA showed a significant effect of treatment groups [*F*_5,24_ = 388, *P* = 0], sampling period [*F*_8,192_ = 433, *P* = 0], and the interaction between treatment groups and sampling period [*F*_40,192_ = 137, *P* = 0].

The selective adenosine A2A receptor antagonist KW 6002 at both doses 1.25 and 2.5 mg/kg significantly (*P* = 0.0001) increased the effect of MDMA (40 mg/kg) on extracellular 3-MT level (Fig. 3d). Repeated measures ANOVA showed a significant effect of treatment groups [*F*_5,28_ = 556, *P* = 0], sampling period [*F*_8,224_ = 215, *P* = 0], and the interaction between treatment groups and sampling period [*F*_40,224_ = 61, *P* = 0]. The basal extracellular 3-MT level in dialysates from the mouse striatum was 14.15 ± 0.95 (pg/10 μl, *n* = 102) and no significant differences between experimental groups were observed.

### The Effect of Caffeine and Adenosine A1 and A2A Receptor Antagonists DPCPX and KW 6002 on MDMA-Induced Changes in Extracellular Level of DOPAC, HVA, and 5-HIAA in the Striatum of the Mouse

**Fig. 4:**
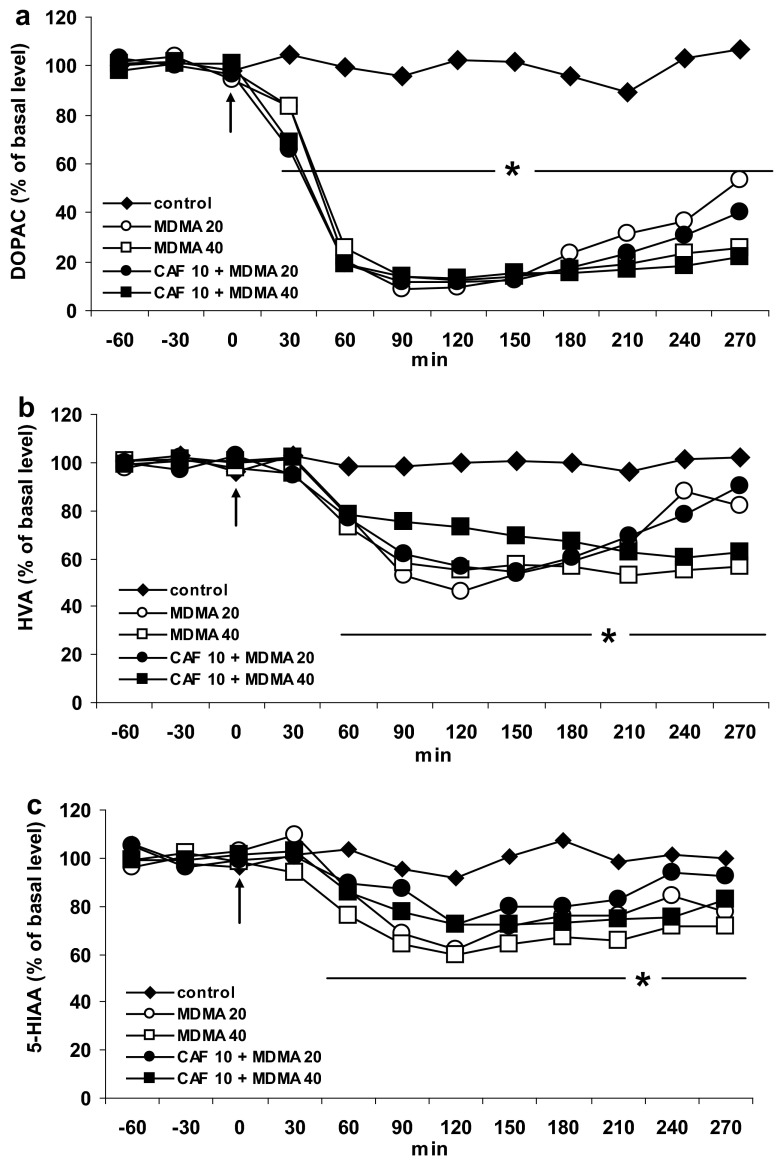
The effect of caffeine (CAF) on changes in extracellular level of DOPAC, HVA, and 5-HIAA induced by MDMA in the mouse striatum. CAF (10 mg/kg) was injected simultaneously with MDMA 20 or 40 mg/kg as indicated with an *arrow*. Values are the mean ± SEM (*n* = 6–8 animals). *P < 0.0001 represents a significant difference in comparison to control group (repeated measures ANOVA and Tukey’s post-hoc test)

MDMA at doses of 20 and 40 mg/kg significantly (*P* = 0.0001) decreased the level of the intraneuronal metabolite of DA, DOPAC to ca. 14 and 9 % of basal level at 120 min after administration. Caffeine at a dose of 10 mg/kg had no effect on the decrease in DOPAC level induced by both doses of MDMA (Fig. 4a). Repeated measures ANOVA of these data showed a statistically significant effect of treatment groups [*F*_5,29_ = 123, *P* = 0], sampling period [*F*_8,232_ = 80, *P* = 0], and the interaction between treatment groups and sampling period [*F*_40,232_ = 9.7, *P* = 0].

**Fig. 5:**
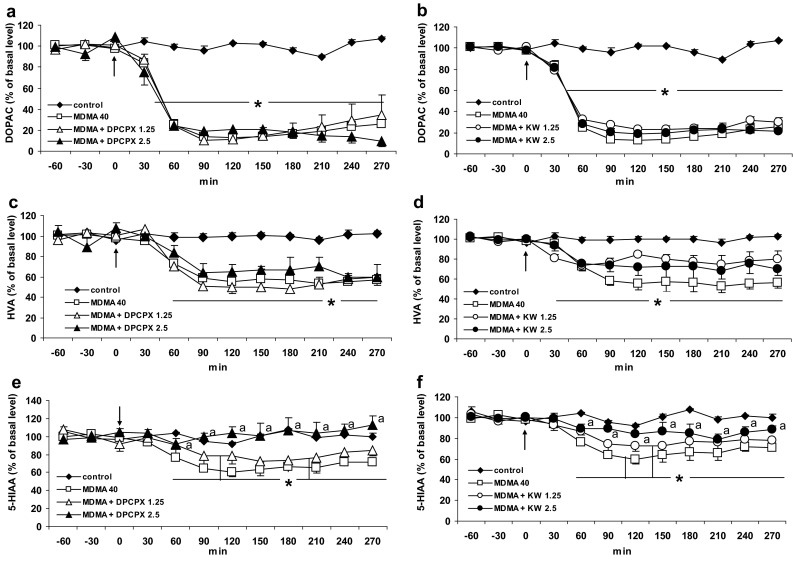
The effect of adenosine A1 and A2A receptor antagonists, DPCPX and KW 6002 (KW) on the changes in extracellular level of DOPAC, HVA, and 5-HIAA induced by MDMA in the mouse striatum. DPCPX (1.25 and 2.5 mg/kg) and KW (1.25 and 2.5 mg/kg) were injected simultaneously with MDMA 40 mg/kg as indicated with an *arrow*. Values are the mean ± SEM (*n* = 6–8 animals). *P < 0.01–0.0002 represents a significant difference in comparison to control group; “*a*” *P* < 0.03–0.0003 represents a significant difference in comparison to MDMA group (repeated measures ANOVA and Tukey’s post-hoc test)

The selective adenosine A1 receptor antagonist DPCPX at both doses, 1.25 and 2.5 mg/kg did not affect the decrease in extracellular DOPAC level produced by MDMA 40 mg/kg (Fig. 5a). Repeated measures ANOVA of these data showed a statistically significant effect of treatment groups [*F*_3,15_ = 77, *P* = 0], sampling period [*F*_8,120_ = 78, *P* = 0], and the interaction between treatment groups and sampling period [*F*_24,120_ = 10.1, *P* = 0]. Similarly, the selective adenosine A2A receptor antagonist KW 6002 at both doses 1.25 and 2.5 mg/kg also did not influence the extracellular level of DOPAC decreased by MDMA (Fig. 5b). Repeated measures ANOVA of these data showed a statistically significant effect of treatment groups [*F*_3,19_ = 77, *P* = 0], sampling period [*F*_8,152_ = 89, *P* = 0], and the interaction between treatment groups and sampling period [*F*_24,152_ = 8.78, *P* = 0].

The extracellular level of HVA was significantly decreased by MDMA 20 and 40 mg/kg (*P* = 0.004 and 0.0002, respectively, in comparison to control group). Caffeine (10 mg/kg) did not change the effect of both doses of MDMA on the extracellular HVA level (Fig. 4b). Repeated measures ANOVA of these data showed a statistically significant effect of treatment groups [*F*_5,28_ = 11.2, *P* = 0.0001], sampling period [*F*_8,224_ = 17.7, *P* = 0], and the interaction between treatment groups and sampling period [*F*_40,224_ = 5.30, *P* = 0].

The adenosine A1 receptor antagonist DPCPX at doses of 1.25 and 2.5 mg/kg was without influence on the extracellular level of HVA decreased by MDMA (40 mg/kg) (Fig. 5c). Repeated measures ANOVA of these data showed a statistically significant effect of treatment groups [*F*_3,15_ = 12.2, *P* = 0.003], sampling period [*F*_8,120_ = 31, *P* = 0], and the interaction between treatment groups and sampling period [*F*_24,120_ = 3.64, *P* = 0.0001]. Similarly, the selective adenosine A2A receptor antagonist KW 6002 at both doses 1.25 and 2.5 mg/kg also did not influence extracellular level of HVA decreased by MDMA (Fig. 5d). Repeated measures ANOVA of these data showed a statistically significant effect of treatment groups [*F*_3,19_ = 7.37, *P* = 0.002], sampling period [*F*_8,152_ = 5.7, *P* = 0.0001], and the interaction between treatment groups and sampling period [*F*_24,152_ = 2.19, *P* = 0.002].

MDMA in a dose of 40 mg/kg but not in the lower one 20 mg/kg significantly decreased extracellular level of serotonin metabolite, 5-HIAA (*P* = 0.008 in comparison to control group). Caffeine (10 mg/kg) had no effect on this decrease (Fig. 4c). Repeated measures ANOVA of these data showed a statistically significant effect of treatment groups [*F*_5,28_ = 5.5, *P* = 0.001], sampling period [*F*_8,224_ = 7.5, *P* = 0.0001], and the interaction between treatment groups and sampling period [*F*_40,224_ = 2.54, *P* = 0.0001].

The adenosine A1 receptor antagonist DPCPX at a dose of 2.5 mg/kg but not at a lower one (1.25 mg/kg) reversed the effect of MDMA (40 mg/kg) on the extracellular 5-HIAA level (*P* = 0.03, Fig. 5c). Repeated measures ANOVA of these data showed a statistically significant effect of treatment groups [*F*_3,15_ = 15.1, *P* = 0.0001], sampling period [*F*_8,120_ = 4.34, *P* = 0.0001], and the interaction between treatment groups and sampling period [*F*_24,120_ = 3.18, *P* = 0.0001]. The decrease in extracellular 5-HIAA level produced by MDMA 40 mg/kg was also counteracted by a higher dose (2.5 mg/kg) of the adenosine A2A receptor antagonist KW 6002 (*P* = 0.0003, Fig. 5f). Repeated measures ANOVA of these data showed a statistically significant effect of treatment groups [*F*_3,19_ = 6.2, *P* = 0.004], sampling period [*F*_8,152_ = 8.21, *P* = 0], but there were no interactions between treatment groups and sampling period [*F*_24,152_ = 1.22, *P* = 0.24].

The basal extracellular levels of DOPAC, HVA, and 5-HIAA (in pg/10 μl, *n* = 183) were 1470 ± 148, 1350 ± 166, and 342 ± 30, respectively, and no significant differences between experimental groups were observed.

### The Effect of Caffeine and Adenosine A1 and A2A Receptor Antagonists DPCPX and KW 6002 on Extracellular Level of DA, 5-HT, 3-MT, DOPAC, HVA, and 5-HIAA in the Striatum of the Mouse

**Fig. 6:**
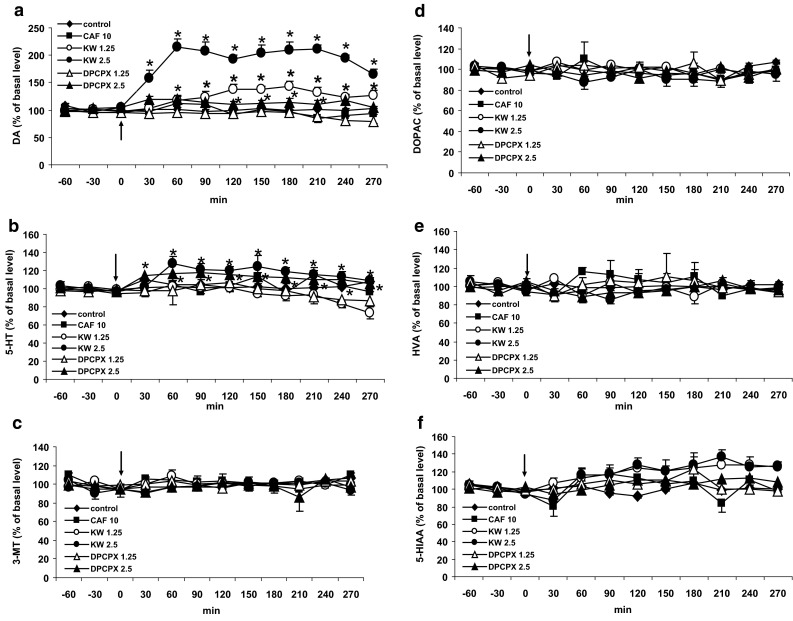
The effect of caffeine (CAF) and adenosine A1 and A2A receptor antagonists, DPCPX and KW 6002 (KW) on extracellular level of DA, 5-HT, 3-MT, DOPAC, HVA, and 5-HIAA in the mouse striatum. CAF (10 mg/kg), DPCPX (1.25 and 2.5 mg/kg), and KW (1.25 and 2.5 mg/kg) were injected as indicated with an *arrow*. Values are the mean ± SEM (*n* = 6–8 animals). *P < 0.05–0.0002 represents a significant difference in comparison to control group (repeated measures ANOVA and Tukey’s post-hoc test)

Caffeine (10 mg/kg) and the adenosine A1 receptor antagonist DPCPX at a lower dose (1.25 mg/kg) given alone were without effect on extracellular level of DA in the mouse striatum (Fig. 6a). However, the adenosine A2A receptor antagonist KW 6002 at both doses (1.25 and 2.5 mg/kg) and the adenosine A1 receptor antagonist DPCPX at a higher dose of 2.5 mg/kg significantly increased extracellular DA level (*P* = 0.0001 and *P* = 0.001, respectively, Fig. 6a). Repeated measures ANOVA of these data showed a statistically significant effect of treatment groups [*F*_5,21_ = 357, *P* = 0], sampling period [*F*_8,168_ = 11.2, *P* = 0], and the interaction between treatment groups and sampling period [*F*_40,168_ = 5.43, *P* = 0].

Extracellular serotonin level was not changed by caffeine but was increased by higher doses (2.5 mg/kg) of the adenosine A1 and A2A receptor antagonists, DPCPX and KW 6002 (Fig. 6b). Repeated measures ANOVA of these data showed no significant effect of treatment groups [*F*_5,18_ = 19.87, *P* = 0.0001], sampling period [*F*_8,144_ = 8.54, *P* = 0], and the interaction between treatment groups and sampling period [*F*_40,144_ = 2.76, *P* = 0.0001].

The level of the extraneuronal DA metabolite, 3-MT was not affected by caffeine and adenosine A1 and A2A receptor antagonists when given alone (Fig. 6c). Repeated measures ANOVA of these data showed a statistically non-significant effect of treatment groups [*F*_5,19_ = 0.69, *P* = 0.64], sampling period [*F*_8,152_ = 1.10, *P* = 0.36], and the interaction between treatment groups and sampling period [*F*_40,152_ = 1.07, *P* = 0.37].

The extracellular level of DOPAC was not changed either by caffeine or the adenosine A1 and A2A receptor antagonists DPCPX and KW 6002, respectively (Fig. 6d). Repeated measures ANOVA of these data showed no significant effect of treatment groups [*F*_5,19_ = 0.60, *P* = 0.7], sampling period [*F*_8,152_ = 0.66, *P* = 0.73], and the interaction between treatment groups and sampling period [*F*_40,152_ = 0.89, *P* = 0.66].

Similarly, the final catecholamine metabolite, HVA was also not affected by caffeine and the adenosine A1 and A2A receptor antagonists DPCPX and KW 6002, respectively (Fig. 6e). Repeated measures ANOVA of these data showed no significant effect of treatment groups [*F*_5,19_ = 0.24, *P* = 0.94], sampling period [*F*_8,152_ = 0.58, *P* = 0.79], and the interaction between treatment groups and sampling period [*F*_40,152_ = 1.13, *P* = 0.3].

Caffeine and the adenosine A1 and A2A receptor antagonists DPCPX and KW 6002 did not influence the extracellular level of the serotonin metabolite, 5-HIAA (Fig. 6f). Repeated measures ANOVA of these data showed no significant effect of treatment groups [*F*_5,19_ = 1.31, *P* = 0.3], sampling period [*F*_8,152_ = 1.89, *P* = 0.06], and the interaction between treatment groups and sampling period [*F*_40,152_ = 1.38, *P* = 0.09].

The basal extracellular levels of DA, 5-HT, 3-MT, DOPAC, HVA, and 5-HIAA (in pg/10 μl, *n* = 78) were 7.6 ± 0.62, 2.1 ± 0.23, 12.7 ± 1.18, 1493 ± 140, 1632 ± 150, and 388 ± 42, respectively, and no significant differences between experimental groups were observed.

### The Effect of Adenosine A1/A2A Receptor Antagonist CGS 15943A on MDMA-Induced Changes in Extracellular Level of DA, 5-HT, 3-MT, DOPAC, HVA, and 5-HIAA in the Striatum of the Mouse

**Fig. 7:**
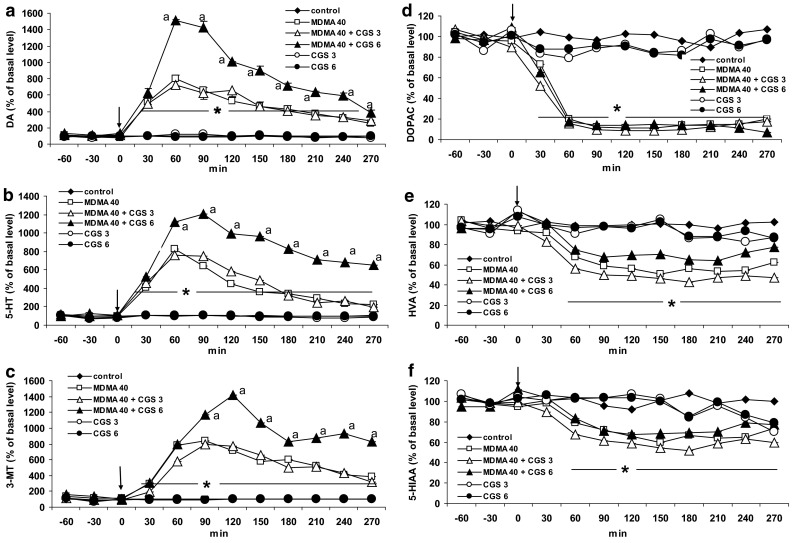
The effect of the adenosine A1/A2A receptor antagonist CGS 15943A (CGS) on MDMA-induced changes in extracellular level of DA, 5-HT, 3-MT, DOPAC, HVA, and 5-HIAA in the mouse striatum. CGS 15943A (3 and 6 mg/kg) was injected simultaneously with MDMA 40 mg/kg as indicated with an *arrow*. Values are the mean ± SEM (*n* = 4 animals). *P < 0.01–0.0002 represents significant difference in comparison to control group; “*a*” *P* < 0.02–0.0002 represents a significant difference in comparison to MDMA group (repeated measures ANOVA and Tukey’s post-hoc test)

The non-selective A1/A2A adenosine receptor antagonist, CGS 15943A at a dose of 6 mg/kg but not at the lower one (3 mg/kg) significantly (*P* = 0.0002) enhanced the increase in striatal DA release induced by MDMA (Fig. 7a). Repeated measures ANOVA of these data showed a significant effect of treatment groups [*F*_5,20_ = 155, *P* = 0], sampling period [*F*_8,160_ = 87, *P* = 0], and the interaction between treatment groups and sampling period [*F*_40,160_ = 24, *P* = 0]. Similarly, CGS 15943A at a higher dose increased 5-HT release (*P* = 0.0002) produced by MDMA (Fig. 7b). Repeated measures ANOVA of these data showed a significant effect of treatment groups [*F*_5,20_ = 156, *P* = 0], sampling period [*F*_8,160_ = 77, *P* = 0], and the interaction between treatment groups and sampling period [*F*_40,160_ = 19, *P* = 0]. The increase in the extraneuronal DA metabolite level, 3-MT was enhanced by CGS 15943A at a dose of 6 mg/kg (Fig. 7c). Repeated measures ANOVA of these data showed a significant effect of treatment groups [*F*_5,20_ = 173, *P* = 0], sampling period [*F*_8,160_ = 38, *P* = 0], and the interaction between treatment groups and sampling period [*F*_40,160_ = 11, *P* = 0]. The level of the intraneuronal metabolite of DA, DOPAC decreased by MDMA was not affected by both doses of CGS 15943A (Fig. 7d). Repeated measures ANOVA of these data showed a significant effect of treatment groups [*F*_5,20_ = 121, *P* = 0], sampling period [*F*_8,160_ = 38, *P* = 0], and the interaction between treatment groups and sampling period [*F*_40,160_ = 11, *P* = 0]. The decrease in HVA induced by MDMA was not affected by CGS 15943A (Fig. 7e). Repeated measures ANOVA of these data showed a significant effect of treatment groups [*F*_5,20_ = 5.92, *P* = 0.002], sampling period [*F*_8,160_ = 12.4, *P* = 0], and the interaction between treatment groups and sampling period [*F*_40,160_ = 2.12, *P* = 0.001]. The serotonin metabolite, 5-HIAA was decreased by MDMA, but its effect was not changed by CGS 15943A (Fig. 7f). Repeated measures ANOVA of these data showed a significant effect of treatment groups [*F*_5,20_ = 7.06, *P* = 0.002], sampling period [*F*_8,160_ = 19.6, *P* = 0], and the interaction between treatment groups and sampling period [*F*_40,160_ = 3.68, *P* = 0]. CGS 15943A given alone did not influence the extracellular level of DA, 5-HT, 3-MT, DOPAC, HVA, and 5-HIAA in the mouse striatum (Fig. 7a–f).

The basal extracellular levels of DA, 5-HT, 3-MT, DOPAC, HVA, and 5-HIAA in this experiment were (in pg/10 μl, *n* = 78) 8.14 ± 1.1, 2.41 ± 0.45, 12.65 ± 1.64, 1615 ± 152, 1299 ± 119, and 337 ± 40, respectively. We did not observe significant differences between experimental groups in the basal levels of neurotransmitters and their metabolites.

## Discussion

The findings of our in vivo study showed that MDMA increased DA and 5-HT release in a dose-dependent manner in the mouse striatum. However, the effect of MDMA on 5-HT release was weaker than that on DA release which does not correspond to the rank order of potency for MDMA inhibition of the DA and 5-HT uptake in vitro, where MDMA exhibited a higher potency at serotonin transporter (SERT) than at dopamine transporter (DAT) (Han and Gu 2006). However, it has to be noted that MDMA has the ability to directly bind to a number of classical neurotransmitter receptors which may contribute to a stronger MDMA effect on DA release. For instance, MDMA by acting directly at brain nicotinic acetylcholine receptors may increase striatal DA release (Faure et al. 2014). In addition, after acute administration of MDMA, DA release may be increased by activation of specific 5-HT receptors. 5-HT2A receptors densely localized on glutamatergic cells in the frontal cortex and activated by the released 5-HT may elicit an increase in glutamate level leading to a rise in DA release in an indirect way (Alex and Pehek 2007). Furthermore, MDMA may suppress nigral GABA release following 5-HT2A/2C receptor activation thus causing disinhibition of the striatal DA neurons (Gudelsky and Yamamoto 2008). The MDMA effect may be additionally strengthened by its major metabolite, 3,4-methylenedioxyamphetamine (MDA) having a higher activity at DAT than at SERT, thereby slightly more increasing DA release than 5-HT (Baumann et al. 2007).

In our study, caffeine alone at a dose of 10 mg/kg failed to alter DA and 5-HT release in the mouse striatum. However, co-administration of caffeine and MDMA had a stronger effect on DA and 5-HT release than that of MDMA alone. It was evidenced that caffeine at doses between 10 and 40 mg/kg increased spontaneous locomotion in mice and rats (Garret and Holtzman 1994; Nikodijeviç et al. 1993). On the other hand, caffeine has been reported to increase DA turnover only at high doses in a manner unrelated to the locomotor stimulation (Morgan and Vestal 1989). Ikeda et al. (2011) reported enhancement of DA release in the striatum of anesthetized rats after concomitant administration of caffeine (20 mg/kg) and MDMA (10 mg/kg). These data are in line with our findings showing in vivo synergistic interaction between caffeine and MDMA in the mouse striatum. The pharmacokinetic interactions have also to be considered in the mechanisms mediating the ability of caffeine to promote MDMA-induced DA release. MDMA and caffeine are metabolized in rodents by the same enzyme, CYP1A2 (Singh et al. 2009). Thus competitive interactions between MDMA and caffeine at CYP1A2 may underlie the mechanism of caffeine/MDMA interaction in their effect on DA as well as 5-HT release. However, MDMA-induced DA and 5-HT release may be also regulated by adenosine receptors as shown in other studies in which agonists of adenosine A1 and A2A receptors decreased methamphetamine-induced DA release or KCl-evoked DA and glutamate release in the rat striatum (Gołembiowska and Żylewska 1997, 1998) and in behavioral studies in which the adenosine A2A agonist CGS 21680 provided a partial protection against MDMA-induced deficit in spatial learning and hippocampal cell death (Kermanian et al. 2012). Caffeine as a non-selective antagonist of adenosine A1 and A2A receptors with in vitro affinity in µM range (Fredholm et al. 1999) may increase DA and glutamate in the striatum via blockade of inhibitory presynaptic adenosine A1 receptors (Borycz et al. 2007; Ciruela et al. 2006; Okada et al. 1996). Similarly, adenosine A1 receptor blockade was shown to increase hippocampal 5-HT release (Okada et al. 1999). A greater response in terms of DA release was observed in the striatal tissue slices superfused with MDMA in combination with caffeine than that obtained following the application of each of the drugs alone (Vanattou-Saïfoudine et al. 2011). Our results show for the first time the effect of the combined treatment of caffeine and MDMA not only on DA but also on 5-HT release as measured by an in vivo microdialysis in mice. We present the evidence that adenosine A1 and A2A receptors are involved in this effect by showing that the selective antagonists of A1 and A2A adenosine receptors, DPCPX and KW 6002 were able to mimic the effect of caffeine. Both adenosine receptor antagonists markedly enhanced MDMA-induced DA and 5-HT release in the mouse striatum, but the potency of their effect was different. The effect of KW 6002 at a lower dose (1.25 mg/kg) was similar to the effect of caffeine co-administered with MDMA 40 mg/kg. However, the effect of a higher dose of KW 6002 (2.5 mg/kg) was much stronger on either DA or 5-HT release than that of caffeine co-administered with MDMA 40 mg/kg. In turn, the effect of the A1 receptor antagonist, DPCPX given at a higher dose (2.5 mg/kg) with MDMA on DA release was weaker in comparison to the effect of combined administration of MDMA with caffeine or a lower dose of KW 6002. Nevertheless, the difference between the group treated with DPCPX (2.5 mg/kg) jointly with MDMA was significant in comparison to MDMA alone. A similar tendency was observed in DPCPX action on 5-HT release. These results indicate that A2A rather than A1 receptor blockade plays a greater role in the potentiating MDMA effect on DA and 5-HT release. One can argue that adenosine receptor antagonists used in this study differ in occupancy of adenosine receptors. However, both KW 6002 and DPCPX show affinity for respective adenosine receptors in a low nM range (Jacobson and Van Rhee 1997; Saki et al. 2013). In our study, both drugs were used in their active doses as evidenced by other tests. DPCPX at doses of 1.25–5 mg/kg increased motility and locomotion in mice (Kuzmin et al. 2006). KW 6002 at doses of 2.5–3 mg/kg increased locomotor activity in mice (Aoyama et al. 2000; Yu et al. 2008) with similar potency to that of caffeine at a dose of 10 mg/kg. Interestingly, our findings showed that KW 6002 and DPCPX alone (but not caffeine) increased the basal extracellular level of DA and 5-HT. These data indicate that DA and 5-HT nerve terminals are under tonic influence of adenosine A1 and A2A receptors. The question arises what mechanism mediates the control of MDMA-induced DA and 5-HT release in the mouse striatum by adenosine receptor blockade. It is known that striatal adenosine A1 receptors have presynaptic and postsynaptic location, and acting as presynaptic heteroreceptors they may modulate neurotransmitter release from DA or 5-HT terminals (Ferré et al. 1997; Vanattou-Saïfoudine et al. 2011). Striatal adenosine A2A receptors are mostly postsynaptic and are highly expressed in striatopallidal GABAergic neurons, where they antagonistically interact with dopamine D2 receptors (Ferré et al. 1997). In addition, they are localized in striatal glutamatergic terminals where they are involved in the modulation of glutamate release (Ciruela et al. 2006). The lack of A2A receptors on striatal DA or 5-HT terminals suggests that the involvement of A2A receptors in the mechanism of DA or 5-HT release may be secondary and related to the changes in the activity of striatal output pathways elicited by A2A postsynaptic receptors, possibly by their negative interaction with dopamine D2 receptors. In fact, there is evidence that peripheral but not local administration of adenosine A2A antagonists increases extracellular concentration of DA in the striatum of naive rats (Gołembiowska et al. 2009; Gołembiowska and Żylewska 1997; Okada et al. 1996). Moreover, behavioral studies indicate that rather A2A than A1 adenosine receptors seem to be involved in the effects of caffeine. Lazarus et al. (2011) using selective gene deletion strategies demonstrated that A2A receptors in the shell region of the nucleus accumbens were responsible for the effect of caffeine on wakefulness. Another study using also genetic knockout models showed that A2A adenosine receptors were required for psychomotor stimulant effect of caffeine in mice (Chen et al. 2010).

Primary action of caffeine seems to be exerted through adenosine A1 and A2A receptors since these receptors bind caffeine at low concentrations (Fredholm et al. 1999). However, caffeine at a dose of 6.25 mg/kg which is known to induce its stimulant motor effects was shown to occupy 66 % of striatal adenosine A2A receptors and further increase in its dose would lead to 55 % occupancy of cortical A1 receptors (Yacoubi et al. 2001). It has to be taken into consideration that caffeine in a dose of 10 mg/kg used in our study in which it significantly increased MDMA-induced DA and 5-HT release may also exert its effect via blockade of adenosine A3 receptor. Caffeine has a low in vitro affinity for A3 receptor (Fredholm et al. 2001). However, under conditions of nearly maximal occupancy of A2A and A1 receptors, adenosine A3 receptor may be responsible for caffeine stimulatory action. A reduction in caffeine-induced stimulation of motor activity in A3R KO mice corroborates this suggestion (Björklund et al. 2008). In spite of a low density of A3 receptors in the brain (Linden 1994) and their ca. 100 fold lower affinity for adenosine in comparison to A1 and A2A receptors (Jacobson and Van Rhee 1997) A3 receptors may be activated under pathological conditions (e.g., ischemia) when adenosine concentration is increased (Chen et al. 2006). However, this is not a case in our study since we did not observe increased adenosine extracellular level after administration of MDMA into mice (results not shown). Therefore, rather A1 and A2A but not A3 adenosine receptors seem to be engaged in the potentiating effect of caffeine on DA and 5-HT release in the mouse striatum.

Another possibility which may explain the effect of caffeine and A2A receptor antagonists on DA and 5-HT release under basal and stimulated conditions is their effect on metabolism of these monoamines. KW 6002 and caffeine represent the class of methylxanthine A2A antagonists which are competitive monoamine oxidase-B (MAO-B) inhibitors (Castagnoli et al. 2003). Thus, methylxanthine A2A antagonists by slowing down cytosolic monoamine utilization and their conversion to DOPAC or 5-HIAA may increase the synaptic level of DA or 5-HT. This effect may be particularly important in the presence of MDMA, thereby increasing the extravesicular monoamine neurotransmitters inside nerve endings which are mainly metabolized by MAO (Sprague et al. 1998). In our study, we observed a decrease in extracellular level of DOPAC, homovanillic acid (HVA), and 5-hydroxyindoleacetic acid (5-HIAA) resulting from the inhibition of DAT or SERT by MDMA. Caffeine or KW 6002 as methylxanthine MAO-B inhibitors should slow down monoamine transformation into DOPAC and HVA, thus additionally causing the decrease in extracellular level of these metabolites. However, in our study, no differences were found in groups treated with the combination of MDMA and KW 6002 or caffeine in comparison to MDMA alone. This may suggest that the effect on MAO-B inhibition by caffeine or KW 6002 is negligible or it is masked by rapid removal of DA from cytosolic compartment and its metabolism in the synaptic cleft. The latter possibility is confirmed by the fact that KW 6002 which inhibits MAO-B with Ki value in µM range (Petzer et al. 2003), but not caffeine which inhibits MAO-B in mM range (Petzer et al. 2013), increased the extracellular DA level when given alone in mice. Interestingly, KW 6002 and DPCPX reversed to control values of the extracellular level of 5-HIAA decreased by MDMA. As serotonin is the substrate for MAO-A isoform (Youdim et al. 2006), increase in extracellular 5-HIAA level may rather result from the stimulation of 5-HT synthesis by these antagonists in the presence of MDMA.

In order to resolve whether the effect of caffeine on MDMA-induced DA and 5-HT release in the mouse striatum is mediated via postsynaptic adenosine A2A receptors but not by MAO inhibition, we tested if the nonxanthine adenosine receptor antagonist CGS 15943A lacking MAO-B activity (Castagnoli et al. 2003) increases, like caffeine and KW 6002, DA and 5-HT release. CGS 15943A is a potent adenosine receptor antagonist with seven-fold greater selectivity for A2A receptor versus A1 receptor, and an IC50 of 3 nM at the A2A receptor (Francis et al. 1988). When used in our study in pharmacologically effective doses (Czuczwar et al. 1991; Griebel et al. 1991), it enhanced MDMA effect on DA and 5-HT release. We also did not observe differences in extracellular level of DOPAC, HVA, and 5-HIAA in groups treated with MDMA alone and its combination with CGS 15943A. These findings suggest that the caffeine effect on MDMA-induced DA and 5-HT release in the mouse striatum is linked to its action on A2A adenosine receptors and is rather not related to the modification of MAO activity.

Interestingly, co-treatment of MDMA and caffeine or adenosine receptor antagonists markedly increased extracellular level of 3-methoxytyramine (3-MT), another metabolite of DA. 3-MT as a product of catabolic enzyme catechol-O-methyltransferase (EC 2.1.1.6; COMT) activity is a good indicator of extraneuronal metabolism of DA released into synaptic cleft. The increase in 3-MT level induced by caffeine or adenosine receptor antagonists co-administered with MDMA suggests that these drugs prolonged DA clearance from extracellular space resulting in a greater sustained extraneuronal DA level. These data strongly indicate that the enhancement of astroglia and microglia reactivity by caffeine (Khairnar et al. 2010) or neuroinflammation (Costa et al. 2014) may be due to an increase in DA release, excessive oxidative stress or formation of serotonergic toxins (Gołembiowska et al. 2009; Gołembiowska and Dziubina 2012; Schmitt and Reith 2010) which all lead to neurotoxicity. On the other hand, caffeine at low doses produces antioxidant effects (Abreu et al. 2011; Gołembiowska et al. 2008, 2013; Górska et al. 2014; Ruiz-Medina et al. 2013; Sinchai et al. 2011) and reduces neurotoxic effect exerted by various toxins. Therefore, further studies especially after prolonged exposure to caffeine and MDMA in animal models are necessary to understand the mechanism of its neurotoxicity.

**Fig. 8:**
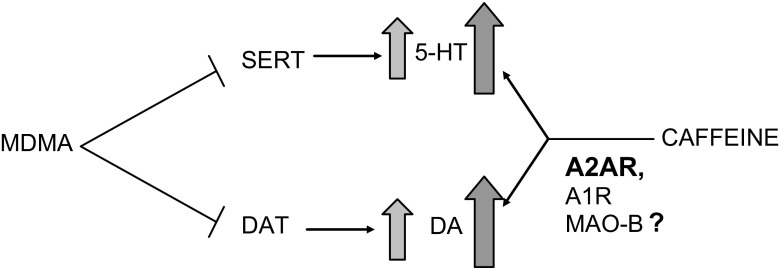
Diagram showing a summary and conclusion of the presented findings. MDMA by blocking 5-HT transporter (SERT) and DA transporter (DAT) produces an increase in extracellular level of 5-HT and DA. Caffeine enhances the effect of MDMA on 5-HT and DA by blocking mainly adenosine A2A receptors and to a smaller extent adenosine A1 receptors. There is no evidence for the role of MAO-B inhibition by caffeine in its effect on monoamine release

In conclusion, the findings of our study demonstrate that caffeine exacerbates the MDMA effect on DA and 5-HT release in the mouse striatum. Its effect is mimicked by selective antagonists of adenosine A1 and A2A receptors corroborating the role of these receptors in the caffeine effect. The summary of our findings is presented in a diagram in Fig. 8. Our neurochemical data may help to understand the mechanism of MDMA neurotoxicity when it is used recreationally in combination with other concomitants, such as caffeine.
